# Impact of renin-angiotensin system targeted therapy on aortic elastic properties assessed by computed tomography

**DOI:** 10.1016/j.ijcha.2024.101562

**Published:** 2024-11-22

**Authors:** Niya Mileva, Panayot Panayotov, Irina Hristova, Greta Koleva, Despina Georgieva, Raya Ivanova, Dobrin Vassilev

**Affiliations:** a Medica Cor Hospital, 1713 Ruse, Bulgaria; b Medical Faculty, Medical University of Sofia, 1431 Sofia, Bulgaria; c Ruse University “Angel Kanchev”, Ruse, Bulgaria; d Department of Cardiology, Pulmonology and Endocrinology, Medical Faculty, Medical University of Pleven, Bulgaria

**Keywords:** Compliance, Distensibility, Hypertension, Aorta

## Abstract

**Background:**

Aortic stiffening is a well-known cardiovascular risk factor. Computed tomography (CT) has proven to be a valuable tool in the assessment of aortic elastic properties. Drugs that inhibit the renin-angiotensin system (RAS) play a central role in cardioprotective therapy. We aimed to evaluate the relationship between aortic elastic properties and RAS-targeted therapy in hypertensive patients.

**Methods:**

This is an observational prospective study of hypertensive patients with nonobstructive coronary artery disease who underwent coronary CT angiography (CCTA). Aortic compliance and distensibility were calculated from the ECG-gated CCTA image. Patients were divided into two groups − those with RAS-targeted therapy − RAS(+) and those with non-RAS-targeted therapy − RAS(−). The elastic properties of the aorta were compared between the two groups.

**Results:**

A total of 142 patients were included in the final analysis. 53.5 % of the population were in the RAS(+) group and 46.5 % in the RAS(−) group. Elastic properties of ascending and descending aorta were significantly higher in the RAS(+) group compared to the RAS(−) group: AA compliance 1.42 ± 0.75 mm^2^/mmHg in the RAS(+) vs 1.03 ± 0.91 mm^2^/mmHg in the RAS(−), p = 0.024; AA distensibility 2.86 ± 1.11 x10^-3^mm^3^ in the RAS(+) vs 1.82 ± 0.97 x10^-3^mm^3^ in RAS(−), p < 0.001; DA compliance 1.45 ± 1.10 mm^2^/mmHg in the RAS(+) vs 1.11 ± 0.91 mm^2^/mmHg in the RAS(−), p 0.031; DA distensibility 2.35 ± 0.84 x10^-3^mm^3^ in the RAS(+) vs 1.73 ± 1.21 x10^-3^mm^3^ in RAS(−), p < 0.001. There was an excellent correlation between RAS therapy and ascending aorta compliance and distensibility (r = 0.901, p < 0.001 and r = 0.875, p < 0.001, respectively).

**Conclusion:**

Patients receiving RAS-blocking treatment revealed significantly higher compliance and distensibility of ascending and descending aorta. In addition, aortic elastic properties were significantly correlated with the RAS-targeted therapy.

## 1 Introduction

Large elastic arteries in the human body have the unique ability to stretch in response to ventricular contractions, acting as a high-compliant elastic buffering chamber. This enables the maintaining of a relatively stable peripheral blood flow during the cardiac cycle [1]. With aging, as a consequence of cumulative exposure to hemodynamic loading, environmental and cardiovascular (CV) risk factors, the large arteries and especially the aorta are prone to losing their elastic properties [2]. The vascular stiffening plays a crucial role in the pathophysiology of arterial hypertension, left ventricular remodeling, heart failure and atherosclerosis. In addition, it may affect CV health as it has been proved to be an independent predictor of mortality, ischemic heart disease and stroke in the general population [3], [4]. In the last decades, various methods have been studied for the evaluation of aortic elastic properties for risk stratification [5], [6], [7], [8], [9]. The advent of computed tomography and the development of modern technologies has made possible the acquisition of high-resolution coronary computed tomography angiography (CCTA) images [10], [11]. It has been reported that aortic distensibility analysis performed using ECG-gated CCTA provided non-invasive, accurate and reproducible measurements [12], [13], [14].

The renin-angiotensin system (RAS) plays a leading regulatory function of cardiovascular function. The RAS system is a physiological pathway functioning through angiotensin receptors that are expressed in different tissues and organs such as myocardium, brain, kidney and vessels [15]. However, despite the normal physiological functions of the RAS, its overactivation may lead to a cascade of inflammatory and oxidative stress processes, that contribute to pathophysiological mechanisms causing cardiovascular diseases [16]. Medications inhibiting the RAS system—i.e., angiotensin converting enzyme inhibitors (ACEi) and angiotensin receptor blockers (ARB), are currently among the first-line treatments for arterial hypertension, heart failure and ischemic heart disease adopted by the contemporary recommendations [17], [18], [19], [20]. To the best of our knowledge, there is no study describing the effect of RAS-targeted therapy on the aortic elastic properties as a CV risk marker. We aimed to evaluate the association of RAS-targeted therapy and the aortic elastic properties obtained through CCTA in patients with arterial hypertension and non-obstructive coronary artery disease.

## 2 Methods

### 2.1 Study population and design

In this observational prospective study, consecutive patients undergoing CCTA for a suspected coronary artery disease with low to moderate risk between September 2022 and June 2023 were considered eligible. Inclusion criteria were: 1) the absence of significant epicardial coronary stenosis (defined as diameter stenosis [DS] > 50 % by visual estimation); 2) patients with known arterial hypertensions on antihypertensive treatment. Patients with previous myocardial infarction (MI), coronary artery bypass graft (CABG) or percutaneous coronary intervention (PCI), left ventricular ejection fraction < 50 %, and acute coronary syndromes were excluded. Patients were divided into two groups depending on the concomitant therapy – RAS (+) for the patients receiving any agent targeting the RAS system and RAS (−) for patients taking non-RAS targeting medical treatment. The protocol was approved by the institutional review board. All patients were managed by the Declaration of Helsinki and provided informed consent for the anonymous publication of scientific data.

### 2.2 Image acquisition

Image acquisition was performed through CCTA using ECG-gated CT scanner (128-slice Somatotom TOP, Siemens, Germany) according to the protocol recommended by the Society of Cardiovascular Computed Tomography [21]. A routine CCTA protocol was performed to determine a scanning range from the tracheal carina to the diaphragm by the calcium score scan. Afterward, 60–80 ml of contrast medium (Iomeron 350 mg/ml, Bracco), according to the patient’s weight, was injected through a dual-head injector into the cubital vein at a rate of 5.0 ml/s, followed by 30 ml of saline solution chaser, using a bolus tracking technique at the slice of aortic root to determine the trigger time. When the density reached a predefined threshold of 90 Hounsfield units (HU), the scan started automatically with a 6-s scan delay during one breath-hold with simultaneous recording of the ECG tracing. The image parameters were slice collimation of 32 × 0.6 mm, and slice acquisition of 128 × 0.6 mm by means of a z-axis flying focal spot, 0.33 s rotation time, with an 83-ms temporal resolution. From the raw data, images were reconstructed every 5 % (5–100 %) of the RR interval with an effective slice thickness of 0.75 mm, an increment of 0.5 mm, and a B26f reconstruction kernel. Blood pressure was measured and recorded at the beginning of the CCTA acquisition. In addition, pulsed waved velocity (PWV) was acquired using Microlife, Watch BP Office Blood pressure monitor.

### 2.3 Aortic elastic properties evaluation

**Fig. 1:**
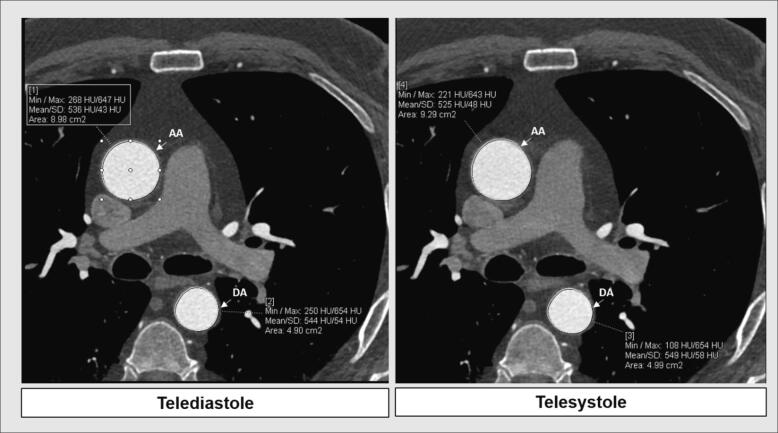
Aortic area measurements from ECG-gated coronary computed tomography angiography images during the cardiac cycle. AA – ascending aorta; DA – descending aorta.

Vessel Analysis software (Syngovia Siemens AG, Germany) was used to quantify the area of ascending and descending aorta through the cardiac cycle. A center line was automatically generated. The cross-sectional area of ROI which was perpendicular to the trace of the vessel was automatically measured at the level of the bifurcation of the pulmonary artery (Fig. 1). Areas of ascending and descending aorta were obtained during telesystole and telediastole detected automatically through the ECG-gated images. Measurements were performed by two blinded independent observers. The average values of the two measurements were filled in as final value.

The following parameters for the evaluation of aortic elastic properties were calculated:

1. Aortic compliance (AC) is the absolute change in area for a given pressure step (P)
C = ΔA/ΔP, mm^2^/mmHg. Where ΔA is the difference between the maximal luminal measured area (As) and the minimal luminal measured area (Ad). ΔP is the difference between systolic pressure (Ps) and diastolic pressure (Pd).
2. Aortic distensibility is defined as the relative change in area at pressure step increase
AD = Δ A/(Ad * Δ P), (AD, ×10^−3^ mmHg^−1^).

#### 2.3.1 Statistics

Data distribution was assessed visually with histograms or with Shapiro-Wilk test as appropriate. Continuous variables with normal distribution were expressed as the mean ± standard deviation and non-normally distributed variables as median and interquartile range. Normal ranges were presented as the 5th and 95th percentiles. Categorical variables were expressed as count and percentages. Differences between groups were analyzed using the *t*-test or the Mann–Whitney *U* test for continuous variables, and the chi-square test or the Fisher’s exact test for categorical variables, as appropriate. Correlation between variables was assessed with either Pearson’s R or Spearman’s ρ as appropriate. Linear and polynomial regression models were fit to assess the relation between continuous variables. Univariate analysis was performed to identify variables associated with increased aortic distensibility. Significant variables were then entered into a multivariable regression analysis model to determine the independent association of each risk factor with increased aortic distensibility. Hazard ratio (HR) and the associated 95 % confidence interval (CI) for each variable were determined. All analyses were performed using R statistical software (R Foundation for Statistical Computing, Vienna, Austria) and Statistical Package for Social Sciences, version 23.0 (SPSS, PC version, Chicago, IL, USA). P value of <0.05 was considered statistically significant.

## 3 Results

### 3.1 Clinical characteristics

**Fig. 2:**
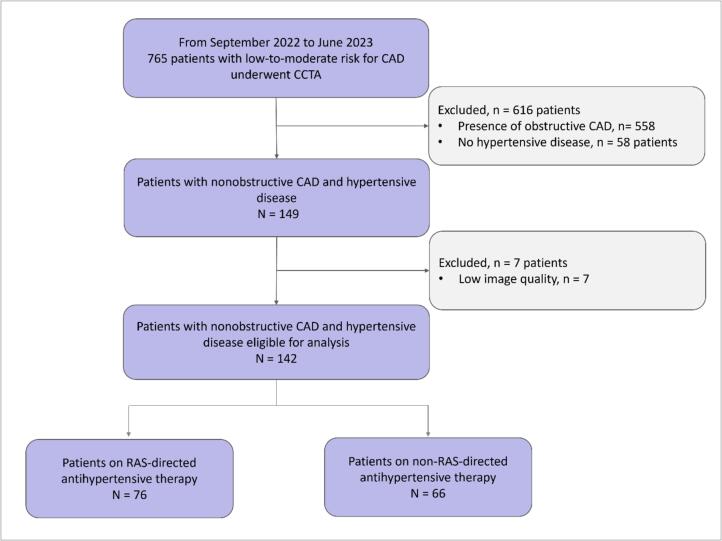
Study Flow Chart. CAD – coronary artery disease; CCTA – coronary computed coronary angiography; RAS – renin angiotensin system.

A total of 765 patients with a low-to-moderate risk for coronary artery disease underwent CCTA between September 2022 and June 2023. As shown in the study flow-chart (Fig. 2), 616 patients were excluded with 558 patients having obstructive CAD and 58 lacking a history of arterial hypertension. Moreover, seven patients were excluded due to inadequate CT image quality, impeding the analysis. The final study population consisted of 142 patients with non-obstructive CAD and hypertensive disease eligible for analysis. 53.5 % (n = 76) of the population were receiving therapy targeted at the RAS system and in 46.5 % (n = 66) of the patients, medical therapy did not include RAS inhibitors.

The baseline and clinical characteristics of the patients are shown in Table 1. In general, the two populations had very similar risk factor profiles. Patients in the RAS (+) group were more frequently males (61 % vs 53 %, p = 0.021), and had a higher prevalence of atrial fibrillation 12 % vs 6 %, p = 0.018 compared to the RAS (−) cohort.

**Table 1:** Demographic and clinical characteristics of the patients included in the study.

**Patient characteristics**	**All** **N = 142**	**RAS (+)** **N = 76**	**RAS (−)** **N = 66**	**P-value**
Age, years (mean ± sd)	57.2 ± 12.1	60.8 ± 11.1	53 ± 12	0.212
Males, n (%)	81 (57)	46 (60.5)	35 (53)	0.021
BMI, mean ± sd	23.3 ± 4.1	24.2 ± 4.1	22.2 ± 3.7	0.063
Family history, n (%)	31 (21.8)	17 (22.0)	14 (21.1)	0.361
Dyslipidemia, n (%)	93 (65.6)	57 (75)	36 (54.5)	0.541
Diabetes, n (%)	26 (18)	16 (21.1)	10 (15.2)	0.753
Chronic kidney disease n (%)	15 (10.6)	8 (10.5)	7 (10.6)	0.817
Cancer, n (%)	2 (1.4)	1 (1.3)	1 (1.5)	0.984
Smoking, n (%)	53 (37)	25 (32.9)	28 (42.4)	0.485
Atrial fibrillation, n (%)	13 (9.2)	9 (12)	4 (6)	0.018
Cerebrovascular disease, n (%)	3 (2.1)	2 (2.6)	1 (1.5)	0.109
Peripheral artery disease, n (%)	4 (2.8)	3 (3.9)	1 (1.5)	0.749
COPD, n (%)	7 (4.9)	3 (3.9)	4 (6.1)	0.262
Beta blocker therapy, n (%)	89 (62.7)	59 (77.6)	30 (45.5)	0.134
Ca-channel blocker, n (%)	51 (35.9)	46 (60.5)	5 (7.6)	0.611
Aldosterone blocker, n (%)	7 (4.9)	3 (3.9)	4 (6.1)	0.062
Central agonist, n (%)	6 (4.2)	3 (3.9)	3 (4.5)	0.275
Diuretic, n (%)	31 (22)	17 (22)	14 (21)	0.482

RAS (+) group – patients receiving renin-angiotensin system-targeted therapy; RAS (−) group − patients receiving non– renin-angiotensin system-targeted therapy; BMI – body mass index; COPD – chronic obstructive pulmonary disease.

### 3.2 CCTA characteristics

**Fig. 3:**
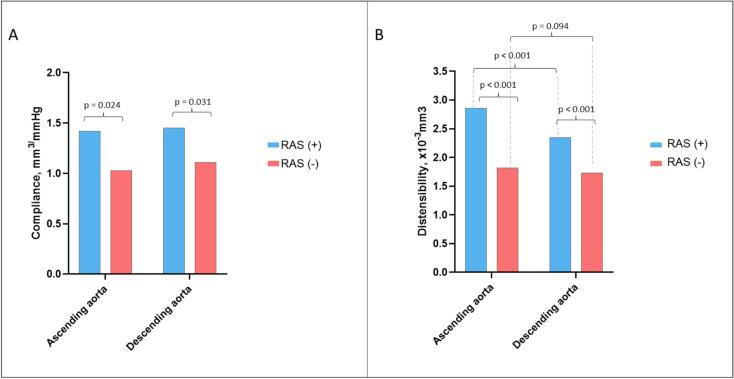
Bar plots illustrating the: A) Compliance of ascending and descending aorta in the RAS (+) and RAS (−) group; B) Distensibility of ascending and descending aorta in the RAS (+) and RAS (−) group. RAS (+) group – patients receiving renin-angiotensin system-targeted therapy. RAS (−) group − patients receiving non– renin-angiotensin system-targeted therapy.

The echocardiographic and CT characteristics of both groups are summarized in Table 2. LV ejection fraction was similar between the two groups. In the RAS (+) cohort, E/e’ ratio was significantly lower (9.2 ± 3.3 vs 10.9 ± 3.7, p = 0.007) compared to the RAS (−) group. Patients treated with RAS-targeted therapy had lower values of PWV (6.69 ± 1.81 m/sec) when compared to the RAS (−) group (7.40 ± 1.33 m/sec, p < 0.001). On the other hand, RAS (+) had a higher Agatston calcium score of 116.8 [25–319] vs 44.9 [12–109] in the RAS (−) group, p = 0.022, and a higher prevalence of aortic sclerosis (18.4 % vs 4.5 % p < 0.001). In addition, both compliance and distensibility of ascending and descending aorta were significantly higher in the RAS (+) group compared to the RAS (−) group. Ascending aorta compliance 1.42 ± 0.75 mm^2^/mmHg in the RAS (+) vs 1.03 ± 0.91 mm^2^/mmHg in the RAS (−), p = 0.024. Ascending aortic distensibility was 2.86 ± 1.11 x10^-3^mm^3^ in the RAS (+) vs 1.82 ± 0.97 x10^-3^mm^3^ in RAS (−), p < 0.001, Fig. 3A. Descending aorta compliance was 1.45 ± 1.10 mm^2^/mmHg in the RAS (+) vs 1.11 ± 0.91 mm^2^/mmHg in the RAS (−), p = 0.031. Descending aortic distensibility was 2.35 ± 0.84 × 10^−3^ mmHg^−1^ in the RAS (+) vs 1.73 ± 1.21 × 10^−3^ mmHg^−1^ in RAS (−), p < 0.001, Fig. 3B.

**Table 2:** CCTA characteristics of the patient population.

**Patient characteristics**	**All** **N = 142**	**RAS (+)** **N = 76**	**RAS (−)** **N = 66**	**P-value**
LV ejection fraction, % (mean ± sd)	56.7 ± 5.7	56.4 ± 5.9	57.2 ± 5.4	0.984
E/e’, mean ± sd	9.8 ± 3.4	9.2 ± 3.3	10.9 ± 3.7	0.007
IVS, mm (mean ± sd)	11.1 ± 1.6	11.8 ± 1.84	10.8 ± 1.50	0.109
LVPW, mm (mean ± sd)	10.6 ± 1.1	10.0 ± 1.33	11.0 ± 1.19	0.114
PWV, m/sec (mean ± sd)	7.21 ± 1.65	6.69 ± 1.81	7.40 ± 1.33	<0.001
Contrast, ml (mean ± sd)	65.0 ± 5.9	63.3 ± 5.8	65.6 ± 6.1	0.707
DLP, (mean ± sd)	750 ± 219	792 ± 202	702.5 ± 230	0.048
Agatston score, median [IQR]	83.4 [12–319]	116.8 [25–319]	44.9 [12–109]	0.022
Likert score, (mean ± sd)	3. 57 ± 0.6	3.56 ± 0.6	3.59 ± 0.6	0.811
CAD-RADS, (mean ± sd)	0.6 ± 0.8	0.6 ± 0.8	0.5 ± 0.8	0.216
High risk plaque, n (%)	2 (1.4)	1 (1.3)	1 (1.5)	0.906
Aortic sclerosis, n (%)	17 (12)	14 (18.4)	3 (4.5)	<0.001
AA compliance, mm^2^/mmHg (mean ± sd)	1.11 ± 0.84	1.42 ± 0.75	1.03 ± 0.91	0.024
AA distensibility, x10^-3^mm3 (mean ± sd)	2.12 ± 0.91	2.86 ± 1.11	1.82 ± 0.97	<0.001
DA compliance, mm^2^/mmHg (mean ± sd)	1.29 ± 1.02	1.45 ± 1.10	1.11 ± 0.91	0.031
DA distensibility, x10^-3^mm^3^ (mean ± sd)	1.96 ± 1.05	2.35 ± 0.84	1.73 ± 1.21	<0.001

RAS (+) group – patients receiving renin-angiotensin system-targeted therapy; RAS (−) group − patients receiving non– renin-angiotensin system-targeted therapy; LV – left ventricular; IVS – interventricular septum; LVPW – left-ventricular posterior wall; PWV – pulsed wave velocity; DLP – dose length product; AA – ascending aorta; DA – descending aorta.

In the RAS (+) group, we observed a preserving of the aortic distensibility gradient – higher distensibility in the proximal ascending aorta followed by a lower distensibility in the descending aorta (2.72 ± 1.03 × 10^−3^ mmHg^−1^ vs 2.35 ± 1.03 × 10^−3^ mmHg^−1^, p < 0.001). In the RAS (−) group, such difference was not observed, mainly due to the lower distensibility in the ascending aorta (1.85 ± 1.09 × 10^−3^ mmHg^−1^ versus 1.79 ± 1.05 × 10^−3^ mmHg^−1^, p = 0.094), Fig. 3B.

**Fig. 4:**
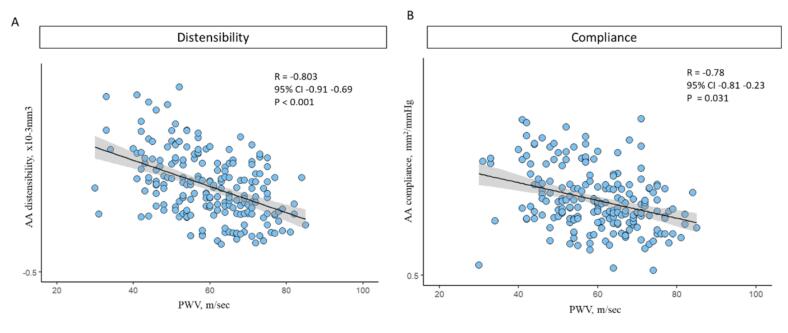
Correlation plots reveal the correlation between: A) ascending aorta distensibility and pulsed wave velocity and B) ascending aorta compliance and pulsed wave velocity. AA − ascending aorta; PWV – pulsed wave velocity.

There was an excellent correlation between RAS therapy and ascending aorta compliance and distensibility (r = 0.901, p < 0.001 and r = 0.875, p < 0.001, respectively). The correlation between descending aorta compliance and distensibility was weaker, however, still statistically significant − (*r* = 0.712, p = 0.021 and *r* = 0.624, p = 0.035, respectively). Additionally, both aortic distensibility and compliance of the ascending aorta were negatively correlated with PWV (r = -0.803, p < 0.001 and *r* = -0.784, p 0.031, respectively), Fig. 4.

Based on univariate regression analysis the following variables were identified as associated with increased AA distensibility – age (HR 0.92, 95 % CI 0.87–0.96); atrial fibrillation (HR 0.97, 95 % CI 0.94–1.00); diabetes (HR 1.09, 95 % CI 1.03–1.14); and RAS-therapy (HR 1.24 95 % CI 1.13––1.57). The only variables that appeared to be independent predictors of increased AA distensibility from the multiregression model were – age (HR 0.90, 95 % CI 0.83–––0.97) and the presence of RAS-therapy (HR 1.35, 95 % CI 1.18–1.56. Table 3.

**Table 3:** Uni- and multi-variate regression analysis for predictors of ascending aortic distensibility.

	**Univariate regression**			**Multivariate regression**		
**Variable**	**HR**	**95 % CI**	**p-value**	**HR**	**95 % CI**	**p-value**
**Age**	**0.92**	**0.87**–**0.96**	**0.021**	**0.90**	**0.83**–**0.97**	**0.019**
**Sex, male**	0.73	0.65–1.29	0.102	−	−	−

**Atrial fibrillation**	**0.97**	**0.94**–**1.00**	**0.08**	0.84	0.65–1.33	0.782
**BMI**	1.13	0.89–1.33	0.236	−	−	−
**Diabetes**	**1.09**	**1.03**–**1.14**	**0.036**	1.03	0.86–1.27	0.339
**RAS therapy**	**1.24**	**1.13**––**1.57**	**0.013**	**1.35**	**1.18**–**1.56**	**0.004**
**BB therapy**	1.11	0.88–1.30	0.623	−	−	−
**Ca-channel blocker**	0.98	0.61–1.45	0.785	−	−	−

BMI – body mass index; RAS – renin-angiotensin system; BB – beta blocker; Ca-channel – calcium channel; HR – hazard ratio; CI – confidence interval.

## 4 Discussion

The present study is the first to evaluate the association between antihypertensive therapy and aortic elastic properties assessed through coronary computed tomography angiography. The main findings of our study are: i) patients receiving RAS-targeted therapy exhibited significantly higher aortic compliance and distensibility in both ascending and descending segments of the aorta when compared to hypertensive patients on non-RAS-targeted therapy. ii) there was a significant correlation between aortic elastic properties and the RAS-targeted therapy; iii)the presence of RAS therapy appeared to be the strongest independent predictor of high AA distensibility; iv) the RAS (+) group exhibited a preserved gradient of aortic distensibility compared to the RAS (−) group.

Previous data have shown that the reduction in cardiovascular risk associated with RAS blockade is due not only to blood pressure control, but also to additional non-hemodynamic effects. (20) One of the essential mechanisms in the protective effect of RAS-inhibiting therapy is that by preventing the binding of angiotensin II to AT1 receptors, the process of enhanced collagen synthesis is inhibited [15]. This mechanism is key to the beneficial effect of RAS blockers on cardiac remodeling and target organ damage [22]. We can hypothesize whether the reduced aortic stiffening in the RAS (+) group is a consequence of a better antihypertensive effect, or it is due to a reduced fibroblast function and less collagen production. Interestingly, the dimensions of the intraventricular septum and LV posterior wall are not significantly different between the two groups. Therefore, the second mechanism discussed above seems more probable.

Although the current study included patients with nonobstructive coronary artery disease, the definition of obstructive CAD used, %DS > 50 %, implies that a proportion of the patients included in the study had some degree of nonobstructive atherosclerosis. Interestingly, patients in the RAS (+) cohort had more pronounced atherosclerosis, with a significantly higher Agatston calcium score and a higher prevalence of aortic sclerosis. However, despite the higher degree of aortic and coronary calcification, the analysis showed greater aortic compliance and distensibility in the RAS (+) cohort. A future analysis evaluating aortic elastic properties in patients with obstructive CAD would be necessary to answer the question of the relationship between RAS therapy, atherosclerosis, and aortic elasticity.

PWV has emerged as a non-invasive and reliable measure of arterial elastic properties and has been shown to be an independent predictor of CV risk [23]. Previously published data have demonstrated a close relationship between PWV and aortic elastic properties [24]. Our results are consistent with these previous data showing that the higher the PWV, the lower the aortic elasticity. However, there are important limitations of noninvasive PWV measurement [25], [26]. By evaluating pulse wave velocity at the extremities, the method does not take into account possible atherosclerosis and stenosis in the large proximal vessels and is therefore prone to inaccuracies [26]. Using CCTA images, we are able to directly evaluate the aortic segments in a non-invasive manner, avoiding potential inaccuracies.

## 5 Limitations

Despite the important findings mentioned, several limitations of the study must be recognized. The single-center observational study design is a methodological limitation regarding the applicability of the study results. Multicenter research on a larger scale is needed to support these findings. Randomized trials would allow for more precise adjustment for risk factors between groups. However, our results did not show significant differences in demographic and clinical characteristics between the two patient cohorts. Given these limitations, the study results remain hypothesis-generating.

## 6 Conclusion

Evaluation of aortic elastic properties in hypertensive patients with nonobstructive CAD was feasible by CCTA. Patients receiving RAS-blocking antihypertensive therapy had significantly higher compliance and distensibility of the ascending and descending aorta. In addition, aortic compliance and distensibility were significantly correlated with RAS-targeted therapy.

## 7 Disclosures

NM reports receiving speaker fees from Abbott, TEVA, Gedeon Richter, and Berlin Chemie. The other authors have nothing to disclose.

## 8 Registration number of clinical studies

31/14.09.2021 – Local Ethical Committee SHATC Medica Cor.

## CRediT authorship contribution statement

**Niya Mileva:** Writing – original draft, Visualization, Formal analysis, Data curation, Conceptualization. **Panayot Panayotov:** Writing – review & editing, Project administration, Data curation. **Irina Hristova:** Writing – review & editing, Validation. **Greta Koleva:** Writing – review & editing, Supervision, Resources. **Despina Georgieva:** Writing – review & editing, Resources. **Raya Ivanova:** Writing – review & editing, Supervision, Methodology. **Dobrin Vassilev:** Writing – review & editing, Supervision, Project administration, Methodology.

## Declaration of competing interest

The authors declare that they have no known competing financial interests or personal relationships that could have appeared to influence the work reported in this paper.
